# Elements of Medical Jurisprudence

**Published:** 1839-07

**Authors:** 


ART. XI.—*Elements of Medical Jurisprudence.* By T. R. BECK, M.D., and J. B. BECK, M.D. *Sixth Edition.* In Two Vols.—*Philadelphia,* 1838. 8vo, pp. 670, 743.

THIS work has been so long before the profession, and has justly acquired so high a character, in Europe as well as in America, that it may seem superfluous to notice it. We are, however, desirous of calling the attention of our younger readers to the best and most complete treatise which is anywhere to be found on this important department of medical science; and we are, at the same time, glad of the opportunity of offering our tribute of respect to the learned and estimable physicians who are its authors. When Mr. Taylor's "Elements" is completed, and we earnestly desire to see the publication of the second volume, the English student will have a work, on a smaller scale, and on a somewhat better plan, which will meet all his wants; but, in the meantime, we earnestly recommend the treatise before us, the present edition of which (the sixth) contains considerable amendments, and many important additions, particularly in the part relating to Persons Found Dead.

